# Health literacy and health behaviors among college students in underdeveloped regions of China: a cross-sectional study in Yunnan Province

**DOI:** 10.3389/fpubh.2026.1883425

**Published:** 2026-07-08

**Authors:** Fengchen Gao, Junqiang Wang, Ying Chen, Xuan Huang, Mengtai Wu, Wenhang Deng, Weiming Li, Yongci Ma, Falei Sun, Yuanyuan Xiao, Qiaoyun Huang

**Affiliations:** 1 School of Public Health, Kunming Medical University, Kunming, Yunnan, China; 2 Department of Epidemiology and Biostatistics, Kunming Medical University, Kunming, Yunnan, China; 3 Academic Affairs Office, Kunming Medical University, Kunming, Yunnan, China; 4 Chenggong District Center for Disease Control and Prevention, Kunming, Yunnan, China; 5 Yunnan Provincial General Practice Training Center, Kunming, Yunnan, China

**Keywords:** college students, health behavior, health literacy, improvement pathways, strategy analysis

## Abstract

**Introduction:**

College students are at a critical stage in the formation and development of their health concepts, lifestyles, and behaviors. Cultivating sound Health Literacy (HL) will have a profound impact on their future health. This study aims to examine the relationship between HL and health behaviors among college students in underdeveloped regions of China, and to propose recommendations and strategies for enhancing HL.

**Methods:**

Based on the National HL Monitoring Survey Questionnaire, a cross-sectional survey was conducted using a self-administered questionnaire in Yunnan. A total of 2,795 participants were included. Exploring strategies to enhance HL through descriptive analysis, multifactor analysis, and mediation effect analysis.

**Results:**

The HL attainment rate among college students in Yunnan Province is 32.92% (95% CI: 31.18–34.66%). Among the three dimensions of HL, Basic Health Skills literacy had the lowest proficiency rate at 23.97%. Among the six categories of health issues, Basic Medical Care literacy, Prevention and Control of Infectious Diseases literacy, and Health Information literacy demonstrated relatively low proficiency rates, at 32.84, 31.20, and 43.15%, respectively. Smoking, habitually stay up late, and breakfast frequency are significantly associated with HL (*p* < 0.05). Basic Health Skills partially mediated the relationship between Basic Health Knowledge and Concepts and Healthy Lifestyle and Behavioral Patterns (*p* < 0.05), with a mediation effect of 28.45%. Health information mediates the transformation of Scientific Concept of Health into the other four issues of HL, with mediation effects accounting for 28.38, 29.67, 26.45 and 25.84%, respectively.

**Conclusion:**

HL is significantly associated with health behaviors. By focusing on health skills and health information, strengthening macro-level policy guidance, and establishing a collaborative network involving individuals, families, schools, and society, the HL of college students in underdeveloped regions can be effectively enhanced.

## Introduction

With China’s rapid socioeconomic development and the growing prominence of public health issues, Health Literacy (HL) has gradually become a focal point of attention across all sectors of society. “HL” refers to an individual’s ability to access and understand basic health information and services, use this information to make informed decisions, and thereby maintain and promote their own health (1). It serves as a key metric for assessing regional health development and directly influences individual health outcomes, including the prevention and management of chronic diseases, as well as the efficiency of health resource utilization (2). Moreover, improving personal HL plays a catalytic role in societal and governmental health governance (3). The 2019 Healthy China Initiative 2019–2030 calls for improving the health education system, popularizing health knowledge, strengthening national health governance, and raising overall HL levels, to achieve a national HL rate of 30% by 2030 (4). While healthy lifestyles are widely recognized to be established during childhood and adolescence (5), the period between ages 18 and 30, particularly during college years, represents a critical window for reinforcing and intervening in individual lifestyle patterns (6). As a population undergoing higher education, guiding college students to acquire scientific and comprehensive health knowledge, enhance their HL, and foster healthy lifestyle habits holds particular significance within higher education. This has profound implications for their future lives.

Research indicates that HL among college students in China generally exceeds that of the general population (7, 8). Yet, significant disparities exist in HL levels among college students across different regions and institutions (9). According to a study by Liu et al. (10), the HL rate among college students in Sichuan Province reached 28.92% in 2021. While, Li’s (11) study indicated that the rate among college students in Tianjin reached 48.2% in 2021. Yang et al. (12) reported Guangzhou’s total HL among enrolled college students at 47.8% in 2020, and Si et al. (13) documented Henan’s college students at 41.3% in 2021. The above evidence indicates that, at any given time, college students’ HL levels are generally higher in economically developed regions than in underdeveloped areas. Yunnan Province, located in China’s southwestern frontier, lags economically compared to central and eastern provinces and is classified as a comprehensively underdeveloped region (14). However, it is home to numerous ethnic minorities and faces significant public health challenges, including major infectious disease prevention and control and endemic diseases, due to its proximity to South and Southeast Asia. Enhancing the HL of its college student population is therefore particularly crucial.

The Knowledge-Attitude-Practice (KAP) theory is a widely applied and well-established cognitive theory in health education research (15). It posits that individuals require sufficient knowledge acquisition and the cultivation of positive beliefs and attitudes to engage in health behaviors. Simultaneously, a bidirectional relationship exists: health behaviors and beliefs within the social environment also promote individuals’ acquisition of health knowledge and development of health beliefs (16, 17). Based on this theory, the foundational knowledge and conceptual literacy within HL may foster the development of health beliefs, enhance health skills, and ultimately promote the adoption of healthy behaviors and lifestyles. Simultaneously, the level of health information literacy can influence various dimensions of HL. Exploring pathways to enhance HL through the multidimensional lens of KAP holds significant importance for improving the HL levels of college students.

**Figure 1:**
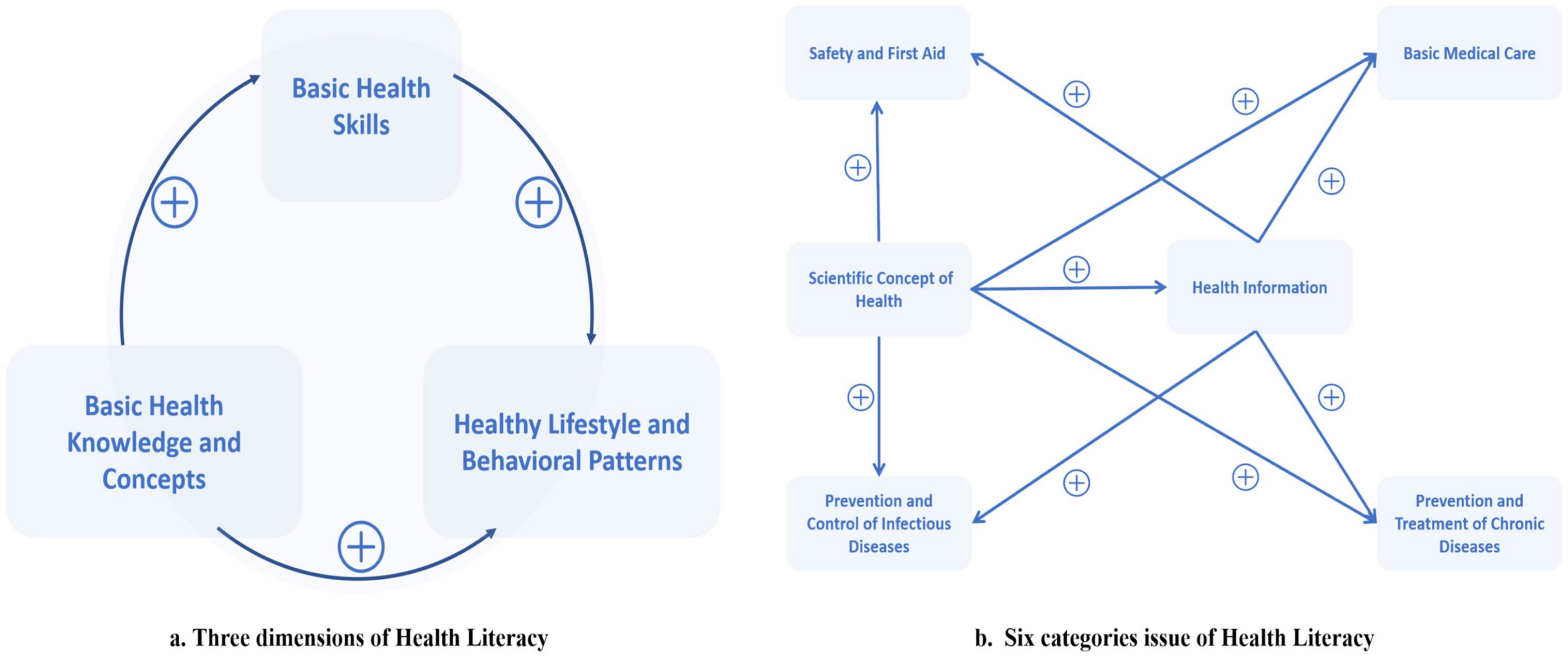
Conceptual model of the relationships among different dimensions of HL based on the KAP theory.

Although existing studies have examined the HL levels and influencing factors among college students across different regions in China (18–20), these investigations have remained limited to descriptive analyses without delving into the relationships among different dimensions of HL. This study aims to understand the current state of HL among college students in underdeveloped regions, represented by Yunnan Province. Based on the KAP theory and Mediator Effect Model, it explores the relationships among different dimensions of HL levels. The study proposed targeted intervention measures and strategies, providing a theoretical basis for the implementation of health education programs in higher education institutions and for policy revisions. Hence, the present study generates the following five research hypotheses (Figure 1):

*H1*: Basic Health Skills serve as a mediator between Basic Health Knowledge and Concepts and Healthy Lifestyle and Behavioral Patterns.

*H2*: Health Information plays a mediating role between Scientific Concept of Health and Prevention and Treatment of Chronic Diseases.

*H3*: Health Information plays a mediating role between Scientific Concept of Health and Prevention and Control of Infectious Diseases.

*H4*: Health Information plays a mediating role between Scientific Concept of Health and Safety and First Aid.

*H5*: Health Information plays a mediating role between Scientific Concept of Health and Basic Medical Care.

## Methods

### Study design and participants

This cross-sectional study surveyed undergraduate students enrolled in higher education institutions in Yunnan Province. Between April 2024 and June 2025, a two-stage random cluster sampling method was employed to select 2,900 participants from 12 universities across Yunnan. The sample encompassed majors in medicine, science, engineering, and liberal arts; undergraduate and vocational programs; and all participants provided informed consent. Data collection utilized either online questionnaires via Wenjuanxing or paper-based surveys based on practical circumstances. Data verification was conducted using inclusion and exclusion criteria.

### Inclusion and exclusion criteria

#### Inclusion criteria

a. Currently enrolled undergraduate students at regular higher education institutions in Yunnan Province; b. Voluntary participation in this study; c. Ability to accurately comprehend the meaning of each questionnaire item.

#### Exclusion criteria

a. Individuals unable to complete the questionnaire due to illness or physical limitations; b. Individuals unable to correctly understand item meanings or exhibiting exclusionary attitudes; c. Completion of the questionnaire showing five consecutive identical responses or logical inconsistencies.

### Sample size estimation

The sample size calculation equation is:

$$N=\frac{\mu_{\alpha }^{2}\times p_{0}(1-p_{0})}{\delta^{2}}\times \text{Deff}$$

Using the 2023 national HL level of 29.70% (21) as a reference, P_0_ = 0.297. With a relative permissible error of *r* = 10%, the absolute permissible error *δ* = P_0_ × 10% = 0.0297. At a 95% confidence level (μα = 1.96), Cluster sampling has lower sampling efficiency, so the design effect (Deff) is set to 1.5. The calculated sample size is 1,363 individuals. Considering a 10% non-response rate, the sample size is further adjusted to 1,515 individuals.

### Survey tool

This study employed a questionnaire developed based on the *National Public Health Literacy Monitoring Survey Questionnaire* (22, 23), supplemented with demographic information and health-related behaviors. China has been conducting health literacy surveys for many years and has achieved significant results; the survey questionnaires have also been widely used among college students (7). The questionnaire comprised two sections: (1) Social Demographic Information and Distribution of Health-Related Behaviors: Including gender, grade, place of origin, parental educational attainment, etc.; daily routines (frequency of weekly exercise, sleep patterns, etc.); unhealthy habits (smoking, excessive drinking, etc.). (2) College Students’ HL Levels: Comprising three dimensions of literacy: Basic Health Knowledge and Concepts (BHKC), Basic Health Skills (BHS),and Healthy Lifestyle and Behavioral Patterns (HLBP) literacy; Covering six categories of health issue literacy: Scientific Concept of Health (SCH), Basic Medical Care (BMC), Safety and First Aid (SFA), Prevention and Control of Infectious Diseases (PCID), Health Information (HI) and Prevention and Treatment of Chronic Diseases (PTCD). This encompasses 56 items organized into 9 dimensions, with a total score of 73 points. The evaluation criterion is that an actual score reaching 80% or above of the total points for all items within a dimension indicates proficiency in that dimension’s HL. The HL questionnaire showed strong internal consistency in the current sample (Cronbach’s *α* = 0.867, Bootstrap 95% CI: 0.859–0.875) as shown in Supplementary Table S1.

## Statistical analysis

Based on the National Health Literacy Monitoring framework, HL scores were calculated according to its defined dimensions and scoring criteria. Categorical data were analyzed using *χ*^2^ tests and continuous data were analyzed using normality tests in SPSS 26.0, with univariate and multivariate analyses performed via binary logistic regression. Forest plots were generated using the `forestplot` package in R 4.5.1. Mediating effect models were constructed with the `lavaan` package. The mediation models were constructed separately, with BHKC as the independent variable, BHS as the mediating variable, and HLBP as the dependent variable; and mediation models were also constructed for the SCH and HI in relation to PCID, PTCD, SFA, and BMC literacy. Model stability was validated through 5,000 bootstrap resamples.

### Quality control

The HL questionnaire adopted by this institute is the national standard monitoring questionnaire, which possesses good reliability and comparability. Before conducting the questionnaire survey, investigators undergo unified training to ensure consistency and accuracy of results across different investigators. To minimize systematic errors in data entry, questionnaires are verified by on-site supervisors before input.

## Results

This study surveyed 2,900 individuals in practice. Due to logical inconsistencies and missing data, 125 invalid questionnaires were excluded. Ultimately, 2,795 valid responses were included, yielding a validity rate of 96.4%.

### General characteristics of the study population

The 2,795 college students included in this survey comprised 984 males (35.2%) and 1,811 females (64.8%); 1,483 were first-year students (53.1%), 589 were second-year students (21.2%), 491 were third-year students (17.6%), and 232 were fourth-year students or above (8.3%); 1,357 were undergraduate students (48.6%), and 1,438 were vocational college students (51.4%); 1,517 students (54.3%) majored in medicine; 2,244 students (80.3%) originated from Yunnan Province; 2,026 students (72.5%) held rural household registration; 1,549 students (55.4%) had parents with junior high school education or below as their highest level of education; most students had monthly living expenses between 1,000 and 1,499 RMB, totaling 1,243 students (44.5%); most students rated their academic performance as average, totaling 1,499 students (53.6%); 2,051 students (73.4%) had received health education.

### Current status of HL among college students in Yunnan Province

**Figure 2:**
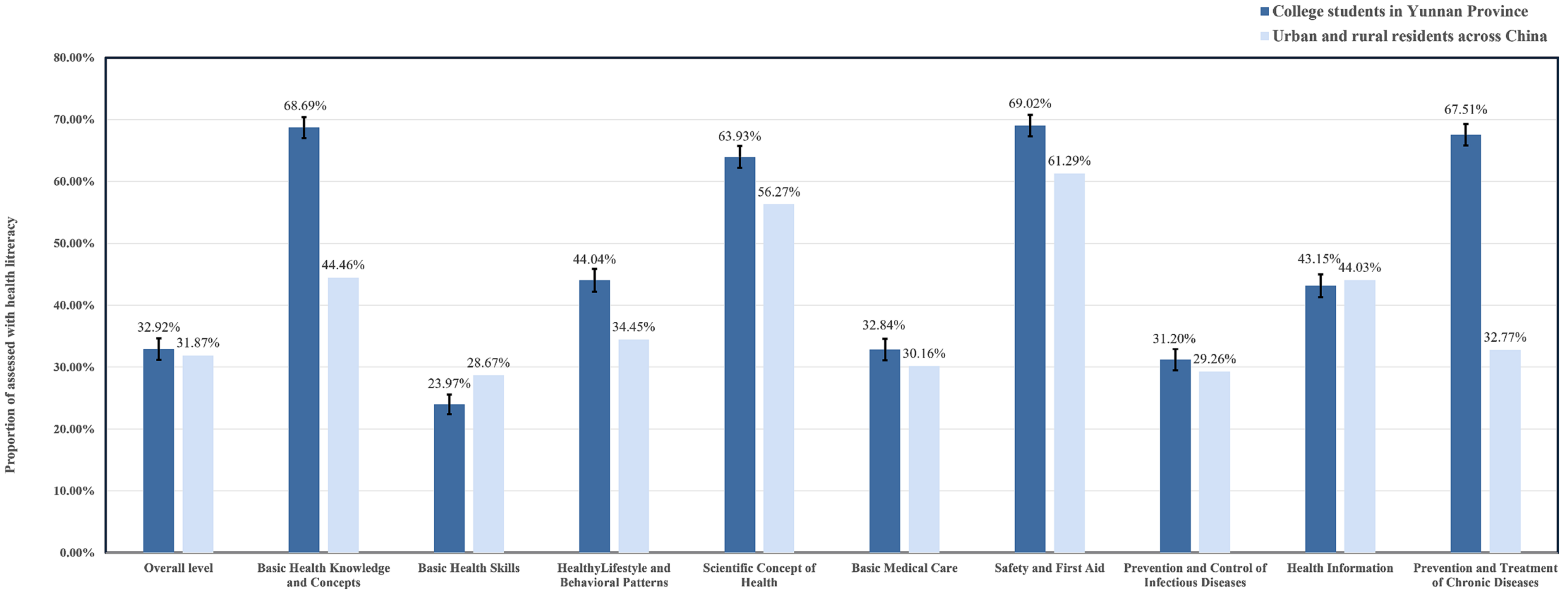
Comparison of HL levels among college students in Yunnan Province and national monitoring levels.

In this study, the HL score of college students in Yunnan Province was 51.41 ± 11.007 points, with a proficiency rate of 32.92% (95% CI: 31.18–34.66%), slightly higher than the national monitoring level of 31.87%. Among the three dimensions of HL, BHKC literacy demonstrated the strongest mastery, with a proficiency rate of 68.69% (95% CI: 66.97–70.41%), significantly exceeding the national monitoring level of 44.46%. BHS literacy showed the weakest mastery, with a proficiency rate of 23.97% (95% CI: 22.39–25.55%), below the national monitoring level of 28.67%. Among the six categories of health issues literacy, SCH, SFA, and PTCD literacy were relatively well mastered, with proficiency rates exceeding 60%. The proficiency rates for BMC and PCID were 32.84% (95% CI: 31.10–34.58%) and 31.20% (95% CI: 29.48–32.92%), respectively, slightly higher than the national monitoring level. HI literacy had a proficiency rate of 43.15% (95% CI: 41.31–44.9%), slightly lower than the national monitoring level of 44.03%. As shown in Supplementary Table S2 and Figure 2.

### Comparison of overall HL levels among different demographic groups

Comparisons between groups revealed that female students demonstrated higher levels of HL than male students (37.2% vs. 25.1%, *p* < 0.001). Significant differences in HL levels were observed across academic years (*p* < 0.001). undergraduate students demonstrated higher HL than vocational college students (46.1% vs. 20.5%, *p* < 0.001); medical majors exhibited higher HL than non-medical majors (42.8% vs. 21.2%, *p* < 0.001); students from Yunnan Province showed lower HL (32.0% vs. 36.7%, *p* = 0.037); HL levels were higher among students with urban household registration than those with rural household registration (41.5% vs. 29.7%, *p* < 0.001); significant differences in HL levels were observed based on parents’ highest educational attainment (*p* = 0.027); HL levels also differed significantly among students with varying monthly living expenses (*p* < 0.001); HL levels showed significant differences among students with different self-rated academic performance levels (*p* < 0.001); students who had received health education demonstrated significantly higher HL levels than those without such training (35.3% vs. 26.3%, *p* < 0.001). In summary, higher HL levels were observed among female students, senior-year students, undergraduate students, medicine majors, students with urban household registration, students with higher parental education levels, students with monthly living expenses between 1,500 and 1,999 RMB, students self-evaluating their academic performance as average or above average, and students who had received health training. As shown in Table 1.

**Table 1:** Comparison of the overall HL levels among people with different demographic characteristics.

Covariates	Total *n* = 2,795 (%)	Without HL *n* = 1,875 (67.08%)	With HL *n* = 920 (32.92%)	*χ* ^2^	*p*
Gender				41.996	<0.001
Male	984 (35.2)	737 (74.9)	247 (25.1)		
Female	1,811 (64.8)	1,138 (62.8)	673 (37.2)		
Grade				132.604	<0.001
First year in college	1,483 (53.1)	1,091 (73.6)	392 (26.4)		
Second year in college	589 (21.1)	424 (72.0)	165 (28.0)		
Third year in college	491 (17.6)	246 (50.1)	245 (49.9)		
Fourth year in college and above	232 (8.3)	114 (49.1)	118 (50.9)		
Type of institution				206.287	<0.001
University	1,357 (48.6)	732 (53.9)	625 (46.1)		
Junior college	1,438 (51.4)	1,143 (79.5)	295 (20.5)		
Major				146.246	<0.001
Medicine	1,517 (54.3)	868 (57.2)	649 (42.8)		
Other majors	1,278 (45.7)	1,007 (78.8)	271 (21.2)		
Place of origin				4.358	0.037
Yunnan province	2,244 (80.3)	1,526 (68.0)	718 (32.0)		
Other provinces	551 (19.7)	349 (63.3)	202 (36.7)		
Household registration				35.258	<0.001
Urban	769 (27.5)	450 (58.5)	319 (41.5)		
Rural	2,026 (72.5)	1,425 (70.3)	601 (29.7)		
Parents’ highest education level				10.933	0.027
Junior high school and below	1,549 (55.4)	1,075 (69.4)	474 (30.6)		
Senior high school or specialized secondary school	672 (24.2)	436 (64.9)	236 (35.1)		
Junior college	296 (10.6)	196 (66.2)	100 (33.8)		
Bachelor’s degree	257 (9.2)	155 (60.3)	102 (39.7)		
Post graduate	21 (0.8)	13 (61.9)	8 (38.1)		
Monthly living expenses				53.767	<0.001
<1,000 CNY	466 (16.7)	376 (80.7)	90 (19.3)		
1,000 ~ 1,499 CNY	1,243 (44.5)	828 (66.6)	415 (33.4)		
1,500 ~ 1,999 CNY	713 (25.5)	434 (60.9)	279 (39.1)		
2,000 CNY and above	373 (13.3)	237 (63.5)	136 (36.5)		
Academic performance				19.121	0.001
Excellent	199 (7.1)	142 (71.4)	57 (28.6)		
Above average	675 (24.2)	422 (62.5)	253 (37.5)		
Average	1,499 (53.6)	998 (66.6)	501 (33.4)		
Below average	356 (12.7)	260 (73.0)	96 (27.0)		
Poor	66 (2.4)	53 (80.3)	13 (19.7)		
Received health education				19.831	<0.001
Yes	2,051 (73.4)	1,237 (64.7)	724 (35.3)		
No	744 (26.6)	548 (73.7)	196 (26.3)		

### Multivariate analysis of overall HL levels

Factors influencing group comparisons were treated as independent variables, while the presence of HL served as the dependent variable in binary logistic regression analysis. Compared to male students, female students demonstrated higher HL levels (OR = 1.549, 95% CI: 1.129–1.877, *p* < 0.001); compared with freshmen, upperclassmen demonstrated higher HL (OR = 1.456, 95% CI: 1.143–1.854, *p* = 0.002 and OR = 1.658, 95% CI: 1.209–2.273, p = 0.002); undergraduate students demonstrated higher HL levels (OR = 3.322, 95% CI: 2.701–4.087, p < 0.001); compared to students with urban household registration, those with rural household registration exhibited lower HL levels (OR = 0.700, 95% CI: 0.557–0.881, p = 0.002); compared to students who self-rated their academic performance as poor, those who self-rated their academic performance as above average and average had higher HL levels (OR = 2.140, 95% CI: 1.093–4.192, *p* = 0.026 and OR = 2.060, 95% CI: 0.736–2.168, *p* = 0.032); compared with students whose monthly living expenses were <1,000 yuan, those with monthly living expenses of 1,000–1,499 yuan and 1,500–1,999 yuan had higher HL levels (OR = 1.359, 95% CI: 1.025–1.801, *p* = 0.033 and OR = 1.429, 95% CI: 1.048–1.950, *p* = 0.025) compared to students who had not received health training, those who had received health training demonstrated higher HL levels (OR = 1.753, 95% CI: 1.418–2.168, *p* < 0.001). In summary, gender, grade level, school level, major, household registration status, self-rated academic performance, and receipt of health training all influence HL as shown in Table 2.

**Table 2:** Binary logistic analysis of overall HL levels.

Covariates	*B*	*SB*	*p*	OR (95%CI)
Gender (ref: male)				
Female	0.438	0.098	<0.001	1.549 (1.129, 1.877)
Grade (ref: first year in college)			<0.001	
Second year in college	−0.080	0.122	0.512	0.923 (0.727, 1.172)
Third year in college	0.375	0.123	0.002	1.456 (1.143, 1.854)
Fourth year in college and above	0.505	0.161	0.002	1.658 (1.209, 2.273)
Type of institution (ref: junior college)				
University	1.201	0.106	<0.001	3.322 (2.701, 4.087)
Major (ref: other majors)				
Medicine	1.002	0.098	<0.001	2.757 (2.251, 3.229)
Place of origin (ref: Yunnan Province)				
Other provinces	0.053	0.113	0.638	1.055 (0.845, 1.317)
Household registration (ref: urban)				
Rural	−0.356	0.117	0.002	0.700 (0.557, 0.881)
Parents’ highest education level (ref: junior high school and below)			0.387	
Senior high school or specialized secondary school	−0.044	0.114	0.701	0.957 (0.766, 1.196)
Junior college	−0.219	0.162	0.175	0.803 (0.585, 1.102)
Bachelor’s degree	−0.304	0.175	0.083	0.738 (0.523, 1.040)
Post graduate	−0.412	0.507	0.417	0.663 (0.245, 1.791)
Monthly living expenses (ref: <1,000 CNY)			0.076	
1,000 ~ 1,499 CNY	0.307	0.144	0.033	1.359 (1.025, 1.801)
1,500 ~ 1,999 CNY	0.357	0.158	0.024	1.429 (1.048, 1.950)
2,000 CNY and above	0.145	0.188	0.439	1.156 (0.800, 1.671)
Scholastic attainment (ref: poor)			0.024	
Excellent	0.518	0.373	0.164	1.679 (0.809, 3.488)
Above average	0.761	0.343	0.026	2.140 (1.093, 4.192)
Average	0.723	0.337	0.032	2.060 (1.065, 3.986)
Below average	0.389	0.355	0.273	1.476 (0.736, 2.961)
Received health education (ref: no)				
Yes	0.562	0.108	<0.001	1.753 (1.418, 2.168)

### Association between health-related behavior distribution and HL

**Figure 3:**
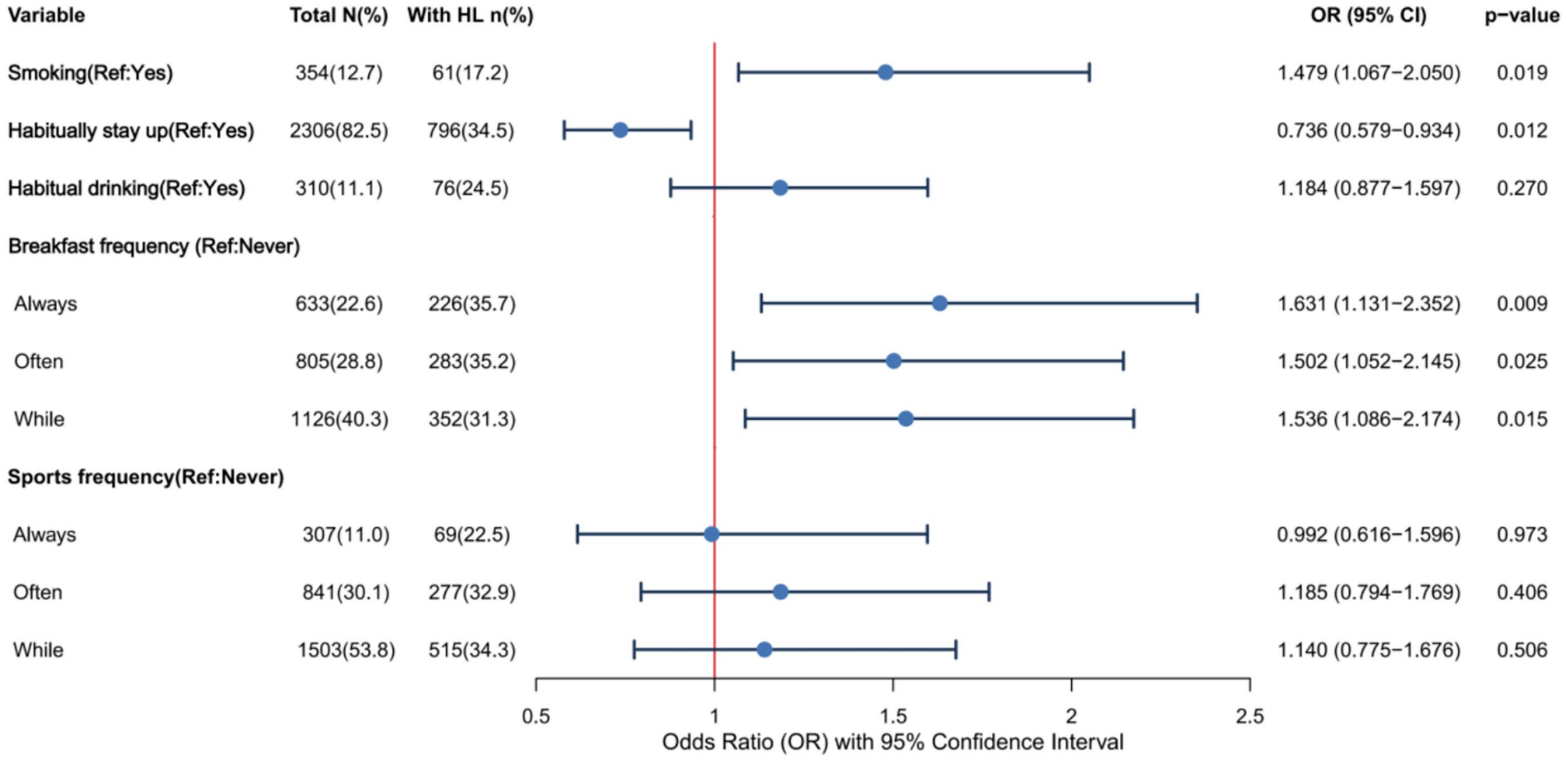
Forest plot of the association between HL and health behaviors. *a, smoking (yes): smoked more than 100 cigarettes cumulatively and still smoking; b, Habitually stay up late (yes): average at least 4 days per week going to bed after 11:00 p.m.; c, habitual drinking: drinking alcohol at least once a week; d, breakfast frequency: always (stick to eating breakfast every day), often (5–6 times per week), while (1–4 times per week); e, sports frequency: always (≥6 times per week), often (3–5 times per week), while (1–2 times per week).

Among the 2,795 college students included in the survey, 354 (12.7%) reported smoking, of whom 61 (17.2%) possessed HL; 2,306 (82.5%) habitually stayed up late (sleeping after 11 p.m. at least 4 days per week on average), among whom 796 (34.5%) possessed HL; 310 (11.1%) habitually consumed alcohol (at least once per week), among whom 76 (24.5%) possessed HL; multivariate logistic analyses were conducted, adjusting for gender, major, grade level, and school tier. Independent variables included smoking status, habitual late-night sleep, habitual alcohol consumption, weekly breakfast consumption, and weekly exercise frequency. The dependent variable was HL. Results indicated a significant association between smoking and HL: students lacking HL exhibited higher smoking rates (OR = 1.479, 95% CI: 1.067–2.050, *p* = 0.019). Habitual late sleep was significantly associated with HL, with habitual late sleep being more prevalent among students with HL (OR = 0.736, 95% CI: 0.579–0.934, *p* = 0.012). Following stratified analysis, it was found that the association between habitual late sleep and HL was significant only among students who rated their academic performance as normal (OR = 0.680, 95% CI: 0.507–0.911, *p* = 0.010; Supplementary Table S4); weekly breakfast frequency was significantly associated with HL, with higher HL levels correlating with higher weekly breakfast frequency (OR = 1.631, 95% CI: 1.131–2.352, *p* = 0.009; OR = 1.502, 95% CI: 1.052–2.145, *p* = 0.025; and OR = 1.536, 95% CI: 1.086–2.174, *p* = 0.025) as shown in Figure 3.

### The relationship among three dimensions of HL: the mediating role of BHS

**Figure 4:**
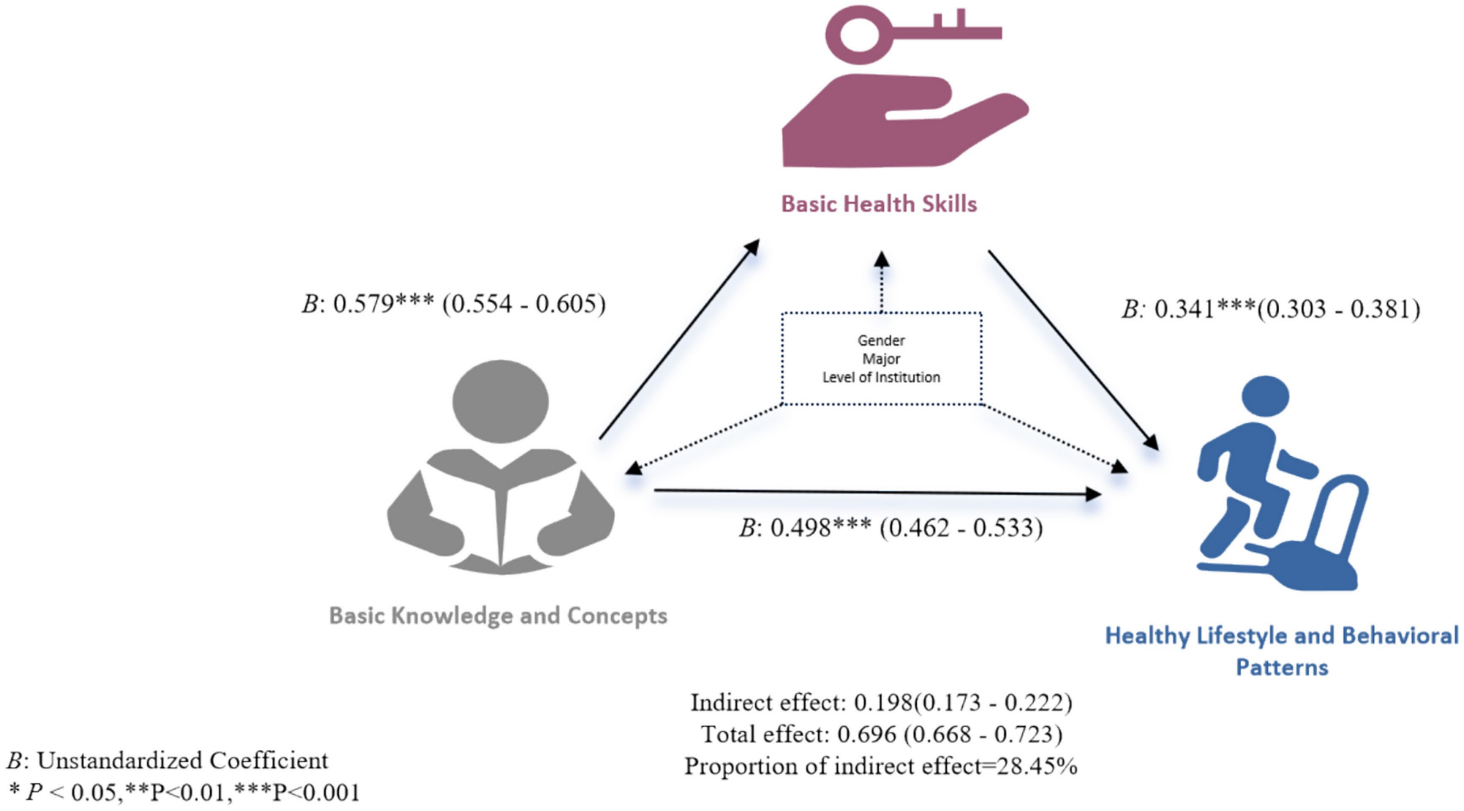
Mediating role of BHS in three dimensions of HL.

The “Knowledge-Belief-Action” model is widely applied in health education. Grounded in knowledge theory, it subtly influences the formation of health beliefs, ultimately leading to action and decisions that promote health. Building upon the “Knowledge-Belief-Action” model and existing research on basic HL, this study hypothesized that BHS mediated the transformation of BHKC into HLBP. Enhancing BHS literacy is expected to improve health-related lifestyle and behavioral competencies. Using BHKC as independent variables, HLBP as dependent variables, and BHS as the mediating variable—while controlling for gender, major, and institution level—a mediation effect model was constructed. Results indicated that BHS partially mediates the relationship between BHKC and HLBP (*p* < 0.05), accounting for 28.45% of the mediating effect as shown in Figure 4 and Supplementary Table S5.

### The relationship among six categories of health issues: the mediating role of HI

**Figure 5:**
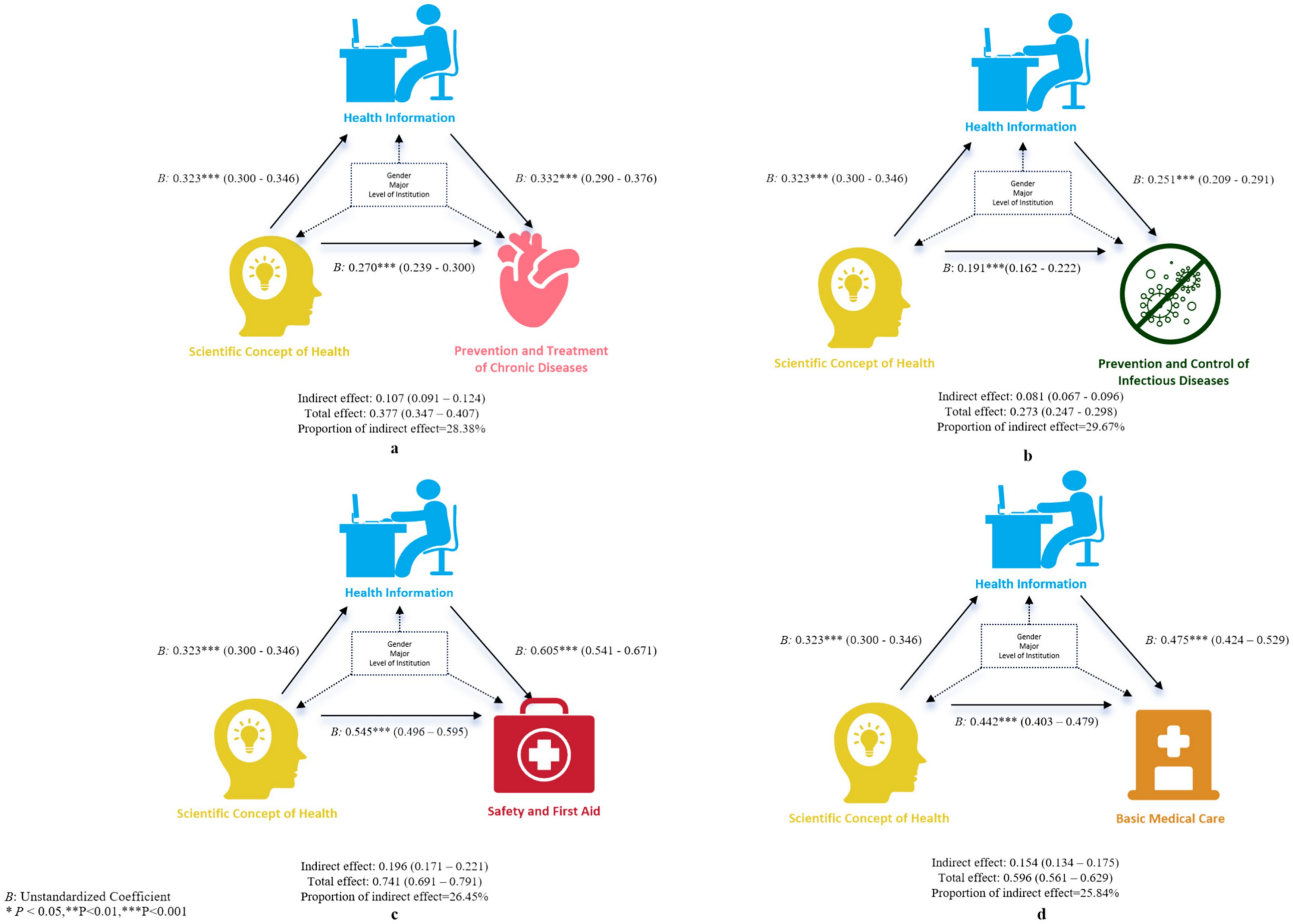
Mediating role of HI in six categories of health issues.

According to the “Knowledge-Belief-Behavior” theory, HI literacy can promote the formation of health behaviors. Based on the comparison between HI literacy and national monitoring levels, we hypothesize that HI mediates the influence of health concepts on HL levels across other dimensions. Using SCH as the independent variable and HI as the mediating variable, we constructed mediation effect models with PTCD, PCID, SFA, and BMC as dependent variables, while controlling for gender, major, and institution level. HI demonstrated partial mediating effects in all models with PTCD, PCID, SFA, and BMC as dependent variables (*p* < 0.05). The respective proportions of mediating effects were 28.38, 29.67, 26.45 and 25.84%, shown in Figure 5 and Supplementary Table S6.

## Discussion

### HL levels and influencing factors among college students

The overall HL level among college students in Yunnan Province stands at 32.92%, exceeding both the national HL level for Chinese residents in 2024 (31.87%) (24)and the HL level for Yunnan residents (29.87%) (25), surpassing the 2023 HL level of 27.2% among college students in Guizhou Province (26). However, it falls below the 2023 HL level of 40.17% among college students in Xi’an (27), the 2021 HL level of 48.2% among college students in Tianjin (11), and the 2018 HL level of 64.4% among college students in Shandong Province (28). This indicates significant regional disparities in college students’ HL levels, potentially linked to local socioeconomic development. Eastern and central regions exhibit greater economic advancement than western areas, with superior cultural and healthcare infrastructure. Students in these regions have greater access to health knowledge and theories, higher healthcare service utilization rates, and consequently higher HL proficiency. In this study, female students demonstrated higher HL than male students (OR = 1.549, 95% CI: 1.129–1.877), consistent with findings from domestic and international research by Uysal et al. (29) and Li et al. (11). This may stem from women’s greater focus on their physical and mental well-being (30), leading them to more actively participate in health training and lectures to acquire knowledge and skills. Undergraduate students demonstrated higher HL levels (OR = 3.322, 95% CI: 2.701–4.087). This may stem from their stronger independent learning abilities, superior mastery of health knowledge and skills, and more comprehensive health concepts. Additionally, undergraduate institutions often provide students with more diverse health-related courses and interdisciplinary educational resources through their curriculum and teaching approaches, leading to superior performance in health knowledge, information evaluation, and health behaviors (31). Compared to freshmen, upperclassmen demonstrated higher HL levels (OR = 1.456, 95% CI: 1.143–1.854; OR = 1.658, 95% CI: 1.209–2.273). Research indicates that after entering college, students receive education and related training that significantly enhances their health awareness, leading to greater understanding of health knowledge and mastery of health behaviors (32); compared to students with urban household registration, those with rural household registration exhibit lower HL levels (OR = 0.700, 95% CI: 0.557–0.881). This disparity may stem from limited access to HI channels, uneven distribution of health education resources, and socioeconomic status within the family and broader social environment (33). Living expense levels correlate with HL levels, with relatively adequate living expenses facilitating higher HL (OR = 1.359, 95% CI: 1.025–1.801; OR = 1.429, 95% CI: 1.048–1.950). However, excessively high or low living expenses may hinder HL improvement. Lower expenses may limit students’ access to health training, healthcare services, and HI, while excessively high expenses may foster unhealthy habits (34). Students who self-rated their academic performance as above average or average demonstrated higher HL levels (OR = 2.140, 95% CI: 1.093–4.192; OR = 2.060, 95% CI: 0.736–2.168), while no statistically significant difference existed between students who self-rated their academic performance as excellent and those who rated it as poor. Research indicates a positive correlation between HL and academic performance, as higher-achieving students possess stronger critical thinking and learning abilities, leading to better mastery of health knowledge and providing a foundation for developing healthy lifestyle habits (35). Simultaneously, high HL contributes to improved quality of life and promotes stable academic performance (36). However, top-performing students may neglect health aspects; despite possessing solid theoretical knowledge, they may lack the application of health skills in daily life and thus fail to achieve high HL levels. Students who have received health training exhibit higher levels of HL (OR = 1.753, 95% CI: 1.418–2.168). Health training enhances students’ health motivation and sense of responsibility by imparting knowledge on disease prevention, chronic disease management, first aid measures, and HI, thereby fostering sound health habits (9, 37).

In terms of HL across three dimensions, the mastery of BHKC was relatively strong at 68.69%, exceeding the national average of 44.46% for urban and rural residents. The proficiency rate for BHS was 23.97%, lower than the national average of 28.67% for urban and rural residents. The proficiency rate for HLBP was 44.04%, higher than the national average of 34.45%. This indicates that among the three dimensions of HL, college students in Yunnan Province demonstrate strong Knowledge and concepts but weaker skills. This aligns with findings from studies by Liu R and Yang S et al. on college students in Southwest China (10, 26), whereas studies by Yang et al. (12) and Li et al. (11) on students in Tianjin and Guangzhou reported better skill acquisition. This suggests deficiencies in BHS among college students in underdeveloped inland regions of Southwest China. Among the six health issue competencies, SCH, SFA, and PTCD have strong mastery, each exceeding 60% proficiency rates. Conversely, BMC, PCID, and HI literacy showed lower proficiency rates of 32.84, 31.20, and 43.15%, respectively. Notably, HI literacy levels were significantly lower than the 86.3% reported in a 2020 study of Yunnan college students (38), aligning with findings from a study of Sichuan college students (14.56%) (10). This discrepancy may stem from the higher proportion of medical students included in the earlier study (76.6%) (38), aligning with the 14.56% rate observed in Sichuan college students (10). This discrepancy may stem from the higher proportion of medical students (76.6%) included in the earlier study compared to the present research.

### The association between HL and health behaviors among college students

HL levels are closely linked to college students’ health behaviors and lifestyles, with higher HL contributing to the formation of good health habits (39). In this study, students lacking HL exhibited higher smoking rates (OR = 1.479, 95% CI: 1.067–2.050). Related research indicates that children and adolescents with low to moderate HL demonstrate higher smoking rates (40), consistent with the findings of this paper. In this study, college students who engaged in nighttime sleep deprivation exhibited higher HL levels. This may stem from the widespread prevalence of nighttime sleep deprivation among college students (41). Even when students possess HL, they may struggle to translate health knowledge and concepts into actual health behaviors. Research indicates that sleep deprivation is common among adolescents in East Asian countries, with most college students experiencing insufficient sleep (daily sleep duration < 8 h) (42, 43). Stratified analysis revealed that a significant association was found only among students with average academic performance, further indicating that college students exhibit a “Health Optimism Bias” mentality (44). They are aware of the dangers of poor sleep habits but still stay up late, rather than doing it solely because of academic pressure. Furthermore, higher HL levels help college students develop healthy dietary concepts, thereby fostering good breakfast habits (45). This aligns with our study’s finding that students with higher HL levels reported higher weekly breakfast consumption frequency.

### The key role of BHS and HI in overall HL

According to the KAP theory, health knowledge serves as a crucial foundation for maintaining health (46). In this study, health skills partially mediated the pathway from health knowledge and beliefs to enhanced health behaviors and lifestyles. The relatively low BHS literacy rate of only 23.97% indicates that knowledge dissemination alone cannot fully translate into health behaviors. Cultivating health skills is essential to internalize knowledge into practical abilities, ultimately fostering healthy lifestyles (47). HI literacy proficiency rates in this study fell below national population monitoring levels, indicating that Chinese college students exhibit weaker capabilities in gathering and utilizing health information, particularly in identifying misinformation and false information (48). Simultaneously, HI plays a partial mediating role in SCH, PTCD, PCID, SFA, and BMC. Enhancing college students’ HI literacy can improve their ability to identify, acquire, and utilize health knowledge, promote the formation of health concepts, increase the efficiency of converting health knowledge into health behaviors, and enhance the effectiveness of health governance (49, 50).

### Strategies and recommendations for enhancing HL among college students

**Figure 6:**
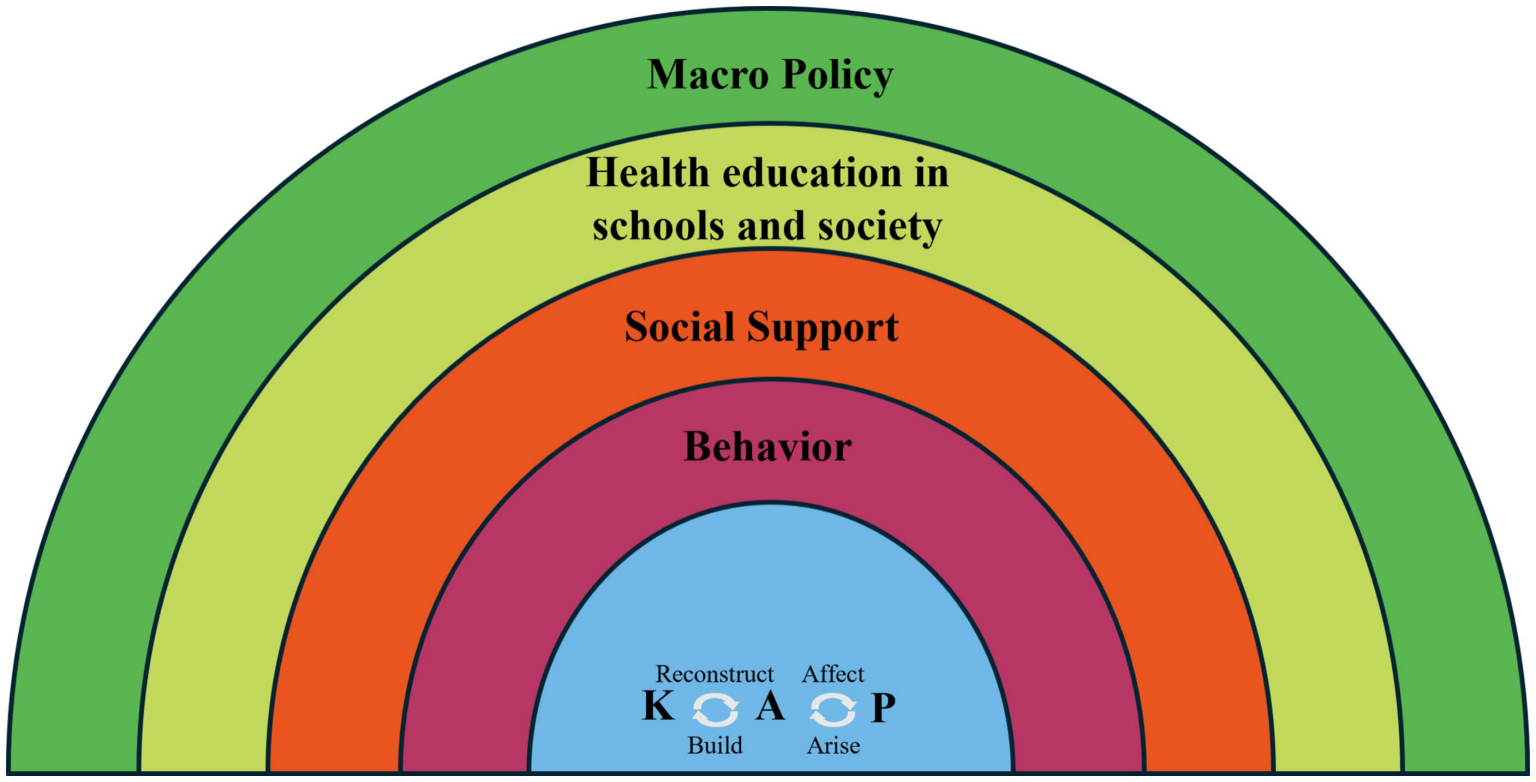
HL enhancement strategy model based on the theory of health determinants.

Health social determinants refer to factors beyond direct causes of disease progression that exert potential impacts on health through individuals’ work and living environments, as well as their social status and resources (51). Dahlgren and Whitehead proposed a five-tiered framework for the social determinants of health: individual factors, individual behaviors and lifestyles, social influences, social structural factors, and the broader societal environment. Factors at the inner layers are influenced by those at the outer layers (52, 53). Based on the theory of social determinants of health and the findings of this study, this research proposed strategies and recommendations for enhancing HL among college students in underdeveloped regions as shown in Figure 6.

### The government’s macro-level policy guidance

The government plays a decisive role in safeguarding public health and enhancing HL nationwide (54) and should formulate specialized policies for college students that align with their age characteristics and local demographic environments. Policies should integrate health education into the higher education system, collaborate with relevant departments to establish quality standards for college health education curricula, and clarify the teaching objectives, content, methods, and assessment approaches for such courses. Furthermore, universities must be mandated to conduct regular health skills training, such as first aid, earthquake preparedness and disaster mitigation, and temporary management of common injuries. Regular supervision and evaluation of health education teaching and related activities should be implemented. Simultaneously, a dedicated fund for college student HL should be established to support campus health promotion and education initiatives. This ensures essential facilities and supporting resources, motivates student participation in health activities, and fosters a campus-wide culture of health. Actively advocate for and promote the participation of all sectors of society in initiatives to enhance college students’ health literacy. Encourage and guide professional health organizations to conduct in-depth, specialized health knowledge and skills training on campus. Through multi-party collaboration, foster a positive social environment, generate powerful synergistic effects, and build a health education collaboration network led by the government and governed collectively by society.

### Integrating school and community health education

As the primary setting for college students’ activities, schools can subtly impart health knowledge and foster health awareness through campus health education, making behavioral and lifestyle changes more achievable (55). Integrating campus health education with community health initiatives creates synergistic effects for enhanced outcomes (56). Institutions should adopt diverse health education approaches, employing interactive teaching methods such as group discussions, presentations, role-playing, and case studies to genuinely boost student engagement and interest (57). Institutions can integrate social network resources—such as collaborating with healthcare organizations, health education agencies, and pharmaceutical companies—to provide students with professional, authoritative health consultations and science-based health knowledge, thereby improving their ability to access and evaluate HI. Simultaneously, incorporating practical components—like participating in health surveys and free clinics—can enhance HL through hands-on experience. Furthermore, schools should leverage their influence to integrate online resources, offering digital health courses, health consultations, and psychological counseling services to broaden access channels for HI.

### Social network support

Families and communities serve as crucial environments for cultivating HL among college students, with family members’ health beliefs and behaviors exerting significant influence on them (58). Parents should utilize health books and authoritative online resources to understand common health issues and knowledge relevant to college students. Engaging in discussions about health topics can bridge communication gaps. Regularly inquiring about their children’s physical and mental well-being, while providing necessary financial and emotional support, enhances college students’ capacity to collaboratively manage health-related pressures. Foster a positive health atmosphere by regularly engaging in family exercise and emphasizing balanced nutrition in meals. Additionally, collaborate with communities and schools to organize health promotion activities, integrating academic expertise, family emotional foundations, and community environmental resources to create a supportive health-promoting environment.

### Enhancing self-health awareness to foster healthy behavioral habits

College students should recognize that health is not merely the absence of disease, but rather a state of complete physical, mental, and social well-being—a dynamic equilibrium resulting from the interaction between biological, psychological, and social factors (59). College students should actively participate in various health lectures, health-related life skills training, and other activities to enhance their health awareness, health management, and problem-solving abilities. Furthermore, leveraging the college platform, they should read health-related books to gain a basic understanding of the human body’s physiological structure and functions. Simultaneously, they should actively seek HI online to improve their health literacy. College students should also actively engage in group activities to strengthen interpersonal communication skills and improve social adaptability. Emotional support and intellectual exchange among peers contribute to mental health and enhance health awareness and concepts. Furthermore, students should assess their own physical and mental health status and undergo regular health checkups to accurately understand their physical condition.

## Limitations

This cross-sectional study examines the current status of health literacy among college students in underdeveloped regions of China, using Yunnan Province as a case study, and proposes strategies and recommendations for improvement. However, due to the limitations of cross-sectional studies, this research can only identify associations between different levels of health literacy but cannot draw causal inferences. Additionally, due to certain constraints, the sample included a relatively high proportion of female students and first-year undergraduates; therefore, caution is warranted when generalizing the findings. Furthermore, questionnaire surveys are inevitably subject to subjective bias during data collection. The involvement of numerous institutions and the lengthy data collection period may affect the timeliness of the cross-sectional study. This study collected data from college students in Yunnan Province. Due to geographical and social differences, conditions may vary across provinces. Further research could collect data in phases from multiple regions to enhance representation, while using longitudinal data to further explore causal relationships.

## Conclusion

This cross-sectional study provides a basic understanding of HL among college students in China’s underdeveloped regions, represented by Yunnan Province. HL among college students in these regions stands at 32.92%, slightly higher than the national average but lower than that of students in coastal and central developed regions. Through government policy guidance, establishing a collaborative network involving individuals, families, schools, and society—based on health knowledge, beliefs, and behaviors—will effectively enhance college students’ HL and contribute to achieving the “Healthy China 2030″ goal.

## Data Availability

The original contributions presented in the study are included in the article/Supplementary material, further inquiries can be directed to the corresponding authors.
