# Supplementary material for: Health literacy and health behaviors among college students in underdeveloped regions of China: a cross-sectional study in Yunnan Province
Source: Front Public Health. 2026 Jul 8;14:1883425. doi: 10.3389/fpubh.2026.1883425 (PMC13388403; doi:10.3389/fpubh.2026.1883425)
Supplement: Supplementary file 1 [file Table_1.DOCX]

**Table S1. The scores of each dimension of HL and their judgment criteria**

| **Content** | **Dimensions** | **Number of items** | **Total score** | **Threshold** |
| --- | --- | --- | --- | --- |
| Health literacy (HL) |  | 56 | 73 | 58 |
| Three aspects of HL | Basic Health Knowledge and Concepts | 20 | 24 | 19 |
|  | Basic Health Skills | 12 | 16 | 13 |
|  | Healthy Lifestyle and Behavioral Patterns | 15 | 20 | 16 |
| Six categories of health issue literacy | Scientific Concept of Health | 8 | 11 | 8 |
|  | Basic Medical Care | 11 | 12 | 10 |
|  | Safety and First Aid | 10 | 14 | 11 |
|  | Prevention and Control of Infectious Diseases | 6 | 7 | 6 |
|  | Health Information | 5 | 6 | 5 |
|  | Prevention and Treatment of Chronic Diseases | 8 | 10 | 8 |

**Table S2. The situation of HL acquisition at different dimensions**

| **Content** | **Dimensions** | **Total score** | **Score**  **Mean (SD)** | **Availability rate (%)** | **95%Confidence Interval (%)** |
| --- | --- | --- | --- | --- | --- |
| Health literacy |  | 73 | 51.41(11.01) | 32.92 | (31.18,34.66) |
| Three aspects of health literacy | Basic Health Knowledge and Concepts | 24 | 19.33(3.81) | 68.69 | (66.97,70.41) |
|  | Basic Health Skills | 16 | 9.98(3.36) | 23.97 | (22.39,25.55) |
|  | Healthy Lifestyle and Behavioral Patterns | 20 | 14.19(3.88) | 44.04 | (42.20,45.88) |
| Six types of health issue literacy | Scientific Concept of Health | 11 | 8.01(2.25) | 63.93 | (62.15,65.71) |
|  | Basic Medical Care | 12 | 8.01(2.49) | 32.84 | (31.10,34.58) |
|  | Safety and First Aid | 14 | 10.99(2.89) | 69.02 | (67.31,70.74) |
|  | Prevention and Control of Infectious Diseases | 7 | 4.59(1.63) | 31.20 | (29.48,32.92) |
|  | Health Information | 6 | 3.95(1.57) | 43.15 | (41.31,44.99) |
|  | Prevention and Treatment of Chronic Diseases | 10 | 7.95(1.83) | 67.51 | (65.77,69.25) |

**Table S3.Test of Normality for Scores on Different Dimensions of HL**

| **Variable** | **Mean** | **SD** | **Skewness** | **Kurtosis** | **K-S Statistic** | ***P*** |
| --- | --- | --- | --- | --- | --- | --- |
| Basic knowledge and concepts | 19.33 | 3.81 | -1.20 | 1.14 | 0.163 | <0.001 |
| Healthy lifestyle and behavioral patterns | 14.19 | 3.88 | -0.73 | -0.02 | 0.124 | <0.001 |
| Basic health skills | 9.98 | 3.36 | -0.57 | -0.16 | 0.115 | <0.001 |
| Scientific concept of health | 8.01 | 2.25 | -0.68 | -0.02 | 0.190 | <0.001 |
| Prevention and control of infectious diseases | 4.59 | 1.63 | -0.25 | -0.61 | 0.127 | <0.001 |
| Prevention and Treatment of chronic diseases | 7.95 | 1.83 | -1.05 | 0.77 | 0.187 | <0.001 |
| Safety and First Aid | 10.99 | 2.89 | -1.34 | 1.26 | 0.197 | <0.001 |
| Basic Medical Care | 8.01 | 2.49 | -0.52 | -0.38 | 0.137 | <0.001 |
| Health Information | 3.95 | 1.57 | -0.52 | -0.54 | 0.180 | <0.001 |

**Table S4. The Association Between Staying Up and HL across different academic performance groups**

| **Covariates** | Academic performance as excellent | | | |  | Academic performance as normal | | | |
| --- | --- | --- | --- | --- | --- | --- | --- | --- | --- |
|  | ***B*** | ***SB*** | ***P*** | ***OR* (95% *CI*)** |  | ***B*** | ***SB*** | ***P*** | ***OR* (95% *CI*)** |
| Gender (Ref: Male) |  |  |  |  |  |  |  |  |  |
| Female | 0.409 | 0.169 | 0.015 | 1.50 (1.081, 2.095) |  | 0.538 | 0.114 | ＜0.001 | 1.712(1.369, 2.142) |
| Type of Institution (Ref: Junior college) |  |  |  |  |  |  |  |  |  |
| University | 1.417 | 0.165 | ＜0.001 | 4.125 (2.984, 5.702) |  | 1.183 | 0.108 | ＜0.001 | 3.246(2.644, 4.030) |
| Major (Ref: Other majors) |  |  |  |  |  |  |  |  |  |
| Medicine | 1.187 | 0.159 | ＜0.001 | 3.277 (2.400, 4.474) |  | 1.123 | 0.112 | ＜0.001 | 3.074(2.468, 3.827) |
| Habitual stay up (Ref: Yes) |  |  |  |  |  |  |  |  |  |
| No | -0.189 | 0.210 | 0.369 | 0.828(0.548, 1.250) |  | -0.386 | 0.149 | 0.010 | 0.680(0.507,0.911) |

* Academic performance as excellent: Academic Performance is rated as “Excellent” or “Above Average”.

Academic performance as normal: Academic Performance is rated as “Average” or “Below Average” or“Poor”.

**Table S5. The Mediating Role of Health Literacy in Three Dimensions**

| **Outcome variable: Healthy lifestyle and behavioral patterns** | B(Unstd.)95% *CI* | ***SE*** | ***Z*** | ***P*** | β (std.)95% *CI* |
| --- | --- | --- | --- | --- | --- |
| Path a：Basic knowledge and concepts → Basic health skills | 0.579 (0.554 - 0.605) | 0.01308 | 44.26481 | <0.001 | 0.657 (0.633 - 0.681) |
| Path b：Basic health skills → Healthy lifestyle and behavioral patterns | 0.341 (0.303 - 0.381) | 0.02012 | 16.95477 | <0.001 | 0.295 (0.261 - 0.329) |
| Path c'：Basic knowledge and concepts → Healthy lifestyle and behavioral patterns | 0.498 (0.462 - 0.533) | 0.01792 | 27.78370 | <0.001 | 0.489 (0.456 - 0.522) |
| Control Variables： |  |  |  |  |  |
| Gender → Basic knowledge and concepts | 1.030 (0.729 - 1.328) | 0.15285 | 6.74059 | <0.001 | 0.129 (0.092 - 0.166) |
| Gender → Basic health skills | -0.088 (-0.283 - 0.109) | 0.09884 | -0.88926 | 0.374 | -0.013 (-0.040 - 0.015) |
| Gender →Healthy lifestyle and behavioral patterns | 0.530 (0.331 - 0.728) | 0.10131 | 5.23046 | <0.001 | 0.065 (0.041 - 0.090) |
| Major → Basic knowledge and concepts | 1.035 (0.765 - 1.309) | 0.13879 | 7.45594 | <0.001 | 0.135 (0.100 - 0.171) |
| Major → Basic health skills | 0.517 (0.333 - 0.697) | 0.09346 | 5.53075 | <0.001 | 0.077 (0.049 - 0.104) |
| Major → Healthy lifestyle and behavioral patterns | 0.305 (0.114 - 0.495) | 0.09780 | 3.11730 | 0.002 | 0.039 (0.015 - 0.064) |
| Level of Institution → Basic knowledge and concepts | 1.904 (1.638 - 2.171) | 0.13460 | 14.14888 | <0.001 | 0.250 (0.216 - 0.283) |
| Level of Institution → Basic health skills | 0.570 (0.383 - 0.756) | 0.09528 | 5.97969 | <0.001 | 0.085 (0.057 - 0.113) |
| Level of Institution →Healthy lifestyle and behavioral patterns | 0.352 (0.153 - 0.557) | 0.10210 | 3.44753 | <0.001 | 0.045 (0.020 - 0.071) |
| **indirect effect** | **0.198 (0.173 - 0.222)** | **0.01270** | **15.55127** | **<0.001** | **0.194 (0.170 - 0.218)** |
| **Direct effect** | **0.498 (0.462 - 0.533)** | **0.01792** | **27.78370** | **<0.001** | **0.489 (0.456 - 0.522)** |
| **Total effect** | **0.696 (0.668 - 0.723)** | **0.01400** | **49.68068** | **<0.001** | **0.683 (0.660 - 0.706)** |

**Table S6. The Mediating Role of Health Literacy on Six Categories of Health Issues**

| **Outcome variable: Prevention and control of infectious diseases** | ***B(Unstd.)95% CI*** | ***SE*** | ***Z*** | ***P*** | ***β (std.)95% CI*** |
| --- | --- | --- | --- | --- | --- |
| Path a：Scientific concept of health → Health information | 0.323 (0.300 - 0.346) | 0.012 | 27.337 | <0.001 | 0.465 (0.433 - 0.496) |
| Path b：Health information → Prevention and control of infectious diseases | 0.251 (0.209 - 0.291) | 0.021 | 12.027 | <0.001 | 0.241 (0.202 - 0.280) |
| Path c' ：Scientific concept of health → Prevention and control of infectious diseases | 0.191 (0.162 - 0.222) | 0.015 | 12.750 | <0.001 | 0.264 (0.224 - 0.304) |
| Control Variables： |  |  |  |  |  |
| Gender → Scientific concept of health | 0.346 (0.165 - 0.522) | 0.091 | 3.802 | <0.001 | 0.073 (0.036 - 0.111) |
| Gender → Health information | 0.201 (0.099 - 0.308) | 0.053 | 3.781 | <0.001 | 0.061 (0.030 - 0.093) |
| Gender →Prevention and control of infectious diseases | 0.381 (0.266 - 0.495) | 0.057 | 6.673 | <0.001 | 0.111 (0.079 - 0.144) |
| Major → Scientific concept of health | 0.532 (0.371 - 0.694) | 0.081 | 6.524 | <0.001 | 0.118 (0.083 - 0.153) |
| Major → Health information | 0.323 (0.226 - 0.423) | 0.050 | 6.419 | <0.001 | 0.103 (0.071 - 0.134) |
| Major →Prevention and control of infectious diseases | 0.281 (0.174 - 0.388) | 0.055 | 5.140 | <0.001 | 0.086 (0.053 - 0.118) |
| Level of Institution → Scientific concept of health | 1.135 (0.974 - 1.297) | 0.082 | 13.869 | <0.001 | 0.252 (0.217 - 0.287) |
| Level of Institution → Health information | 0.383 (0.280 - 0.486) | 0.052 | 7.302 | <0.001 | 0.122 (0.089 - 0.155) |
| Level of Institution →Prevention and control of infectious diseases | 0.125 (0.014 - 0.237) | 0.056 | 2.227 | 0.026 | 0.038 (0.005 - 0.072) |
| **indirect effect** | **0.081 (0.067 - 0.096)** | **0.007** | **10.986** | **<0.001** | **0.112 (0.092 - 0.132)** |
| **Direct effect** | **0.191 (0.162 - 0.222)** | **0.015** | **12.750** | **<0.001** | **0.264 (0.224 - 0.304)** |
| **Total effect** | **0.273 (0.247 - 0.298)** | **0.013** | **21.005** | **<0.001** | **0.376 (0.342 - 0.410)** |
| **Outcome variable: Prevention and treatment of chronic diseases** |  |  |  |  |  |
| Path a：Scientific concept of health → Health information | 0.323 (0.300 - 0.346) | 0.012 | 27.337 | <0.001 | 0.465 (0.433 - 0.496) |
| Path b：Health information → Prevention and treatment of chronic diseases | 0.332 (0.290 - 0.376) | 0.022 | 14.860 | <0.001 | 0.284 (0.248 - 0.321) |
| Path c' ：Scientific concept of health →Prevention and treatment of chronic diseases | 0.270 (0.239 - 0.300) | 0.016 | 17.090 | <0.001 | 0.332 (0.295 - 0.368) |
| Control Variables： |  |  |  |  |  |
| Gender → Scientific concept of health | 0.346 (0.165 - 0.522) | 0.091 | 3.802 | <0.001 | 0.073 (0.036 - 0.111) |
| Gender → Health information | 0.201 (0.099 - 0.308) | 0.053 | 3.781 | <0.001 | 0.061 (0.030 - 0.093) |
| Gender →Prevention and treatment of chronic diseases | 0.223 (0.102 - 0.344) | 0.061 | 3.628 | <0.001 | 0.058 (0.027 - 0.089) |
| Major → Scientific concept of health | 0.532 (0.371 - 0.694) | 0.081 | 6.524 | <0.001 | 0.118 (0.083 - 0.153) |
| Major → Health information | 0.323 (0.226 - 0.423) | 0.050 | 6.419 | <0.001 | 0.103 (0.071 - 0.134) |
| Major →Prevention and treatment of chronic diseases | 0.177 (0.064 - 0.292) | 0.059 | 3.032 | 0.002 | 0.048 (0.017 - 0.079) |
| Level of Institution → Scientific concept of health | 1.135 (0.974 - 1.297) | 0.082 | 13.869 | <0.001 | 0.252 (0.217 - 0.287) |
| Level of Institution → Health information | 0.383 (0.280 - 0.486) | 0.052 | 7.302 | <0.001 | 0.122 (0.089 - 0.155) |
| Level of Institution →Prevention and treatment of chronic diseases | 0.310 (0.189 - 0.430) | 0.060 | 5.12910 | <0.001 | 0.085 (0.052 - 0.117) |
| **indirect effect** | **0.107 (0.091 - 0.124)** | **0.008** | **12.741** | **<0.001** | **0.132 (0.113 - 0.152)** |
| **Direct effect** | **0.270 (0.239 - 0.300)** | **0.016** | **17.090** | **<0.001** | **0.332 (0.295 - 0.368)** |
| **Total effect** | **0.377 (0.347 - 0.407)** | **0.015** | **24.605** | **<0.001** | **0.464 (0.431 - 0.497)** |
| **Outcome variable: Safety and First Aid** |  |  |  |  |  |
| Path a：Scientific concept of health → Health information | 0.323 (0.300 - 0.346) | 0.012 | 27.337 | <0.001 | 0.465 (0.433 - 0.496) |
| Path b：Health information → Safety and First Aid | 0.605 (0.541 - 0.671) | 0.033 | 18.313 | <0.001 | 0.329 (0.295 - 0.362) |
| Path c' ：Scientific concept of health → Safety and First Aid | 0.545 (0.496 - 0.595) | 0.025 | 21.574 | <0.001 | 0.425 (0.390 - 0.460) |
| Control Variables： |  |  |  |  |  |
| Gender → Scientific concept of health | 0.346 (0.165 - 0.522) | 0.091 | 3.802 | <0.001 | 0.073 (0.036 - 0.111) |
| Gender → Health information | 0.201 (0.099 - 0.308) | 0.053 | 3.781 | <0.001 | 0.061 (0.030 - 0.093) |
| Gender →Safety and First Aid | 0.026 (-0.151 - 0.199) | 0.089 | 0.289 | 0.773 | 0.004 (-0.025 - 0.033) |
| Major → Scientific concept of health | 0.532 (0.371 - 0.694) | 0.081 | 6.524 | <0.001 | 0.118 (0.083 - 0.153) |
| Major → Health information | 0.323 (0.226 - 0.423) | 0.050 | 6.419 | <0.001 | 0.103 (0.071 - 0.134) |
| Major →Safety and First Aid | -0.005 (-0.172 - 0.160) | 0.086 | -0.056 | 0.955 | -0.001 (-0.030 - 0.028) |
| Level of Institution → Scientific concept of health | 1.135 (0.974 - 1.297) | 0.082 | 13.869 | <0.001 | 0.252 (0.217 - 0.287) |
| Level of Institution → Health information | 0.383 (0.280 - 0.486) | 0.052 | 7.302 | <0.001 | 0.122 (0.089 - 0.155) |
| Level of Institution →Safety and First Aid | -0.095 (-0.263 - 0.077) | 0.087 | -1.090 | 0.276 | -0.016 (-0.046 - 0.013) |
| **indirect effect** | **0.196 (0.171 - 0.221)** | **0.013** | **15.002** | **<0.001** | **0.153 (0.134 - 0.171)** |
| **Direct effect** | **0.545 (0.496 - 0.595)** | **0.025** | **21.574** | **<0.001** | **0.425 (0.390 - 0.460)** |
| **Total effect** | **0.741 (0.691 - 0.791)** | **0.025** | **29.207** | **<0.001** | **0.578 (0.546 - 0.609)** |
| **Outcome variable: Basic medical care** |  |  |  |  |  |
| Path a：Scientific concept of health → Health information | 0.323 (0.300 - 0.346) | 0.012 | 27.337 | <0.001 | 0.465 (0.433 - 0.496) |
| Path b：Health information → Basic medical care | 0.475 (0.424 - 0.529) | 0.027 | 17.706 | <0.001 | 0.299 (0.267 - 0.332) |
| Path c' ：Scientific concept of health → Basic medical care | 0.442 (0.403 - 0.479) | 0.019 | 23.043 | <0.001 | 0.400 (0.366 - 0.433) |
| Control Variables： |  |  |  |  |  |
| Gender → Scientific concept of health | 0.346 (0.165 - 0.522) | 0.091 | 3.802 | <0.001 | 0.073 (0.036 - 0.111) |
| Gender → Health information | 0.201 (0.099 - 0.308) | 0.053 | 3.781 | <0.001 | 0.061 (0.030 - 0.093) |
| Gender →Basic medical care | 0.446 (0.300 - 0.596) | 0.076 | 5.897 | <0.001 | 0.086 (0.057 - 0.114) |
| Major → Scientific concept of health | 0.532 (0.371 - 0.694) | 0.081 | 6.524 | <0.001 | 0.118 (0.083 - 0.153) |
| Major → Health information | 0.323 (0.226 - 0.423) | 0.050 | 6.419 | <0.001 | 0.103 (0.071 - 0.134) |
| Major →Basic medical care | 0.280 (0.134 - 0.427) | 0.074 | 3.786 | <0.001 | 0.056 (0.027 - 0.085) |
| Level of Institution → Scientific concept of health | 1.135 (0.974 - 1.297) | 0.082 | 13.869 | <0.001 | 0.252 (0.217 - 0.287) |
| Level of Institution → Health information | 0.383 (0.280 - 0.486) | 0.052 | 7.302 | <0.001 | 0.122 (0.089 - 0.155) |
| Level of Institution →Basic medical care | 0.332 (0.188 - 0.484) | 0.075 | 4.415 | <0.001 | 0.067 (0.037 - 0.096) |
| **indirect effect** | **0.154 (0.134 - 0.175)** | **0.010** | **14.736** | **<0.001** | **0.139 (0.121 - 0.157)** |
| **Direct effect** | **0.442 (0.403 - 0.479)** | **0.019** | **23.043** | **<0.001** | **0.400 (0.366 - 0.433)** |
| **Total effect** | **0.596 (0.561 - 0.629)** | **0.018** | **33.980** | **<0.001** | **0.539 (0.510 - 0.568)** |
